# Investigation of SCC*mec* types I–IV in clinical isolates of methicillin-resistant coagulase-negative staphylococci in Ahvaz, Southwest Iran

**DOI:** 10.1042/BSR20200847

**Published:** 2020-05-11

**Authors:** Effat Abbasi Montazeri, Sakineh Seyed-Mohammadi, Aram Asarehzadegan Dezfuli, Azar Dokht Khosravi, Maryam Dastoorpoor, Mitra Roointan, Morteza Saki

**Affiliations:** 1 Infectious and Tropical Diseases Research Center, Health Research Institute, Ahvaz Jundishapur University of Medical Sciences, Ahvaz, Iran; 2 Department of Microbiology, Faculty of Medicine, Ahvaz Jundishapur University of Medical Sciences, Ahvaz, Iran; 3 Student Research Committee, Ahvaz Jundishapur University of Medical Sciences, Ahvaz, Iran; 4 Department of Biostatistics and Epidemiology, Menopause Andropause Research Center, Ahvaz Jundishapur University of Medical Sciences, Ahvaz, Iran

**Keywords:** Coagulase-negative staphylococci, Methicillin resistance, MR-CoNS, SCCmec

## Abstract

Today methicillin resistant coagulase-negative staphylococci (MR-CoNS) are important in terms of causing significant nosocomial infections. Besides, MR-CoNS are confirmed as the reservoir of SCC*mec* elements that carry *mecA* (methicillin-resistant) gene. Hence, the present study was designed to evaluate the susceptibility pattern, prevalence and diversity of SCC*mec* types I, II, III, and IV in MR-CoNS strains. In this cross-sectional study, 44 clinical isolates of MR-CoNS were identified using the cefoxitin disc method and further confirmation by polymerase chain reaction (PCR) amplification of the *mecA* gene. Antimicrobial susceptibility of isolates was investigated by disc diffusion. The identification of CoNS was done by amplification and sequencing of the *tuf* gene. Multiplex PCR method was done for the determination of SCC*mec* types. In the present study, the *Staphylococcus epidermidis* and *Staphylococcus haemolyticus* were the most predominant isolates with a prevalence of 45.4%. The highest resistance rates were observed against erythromycin (84.1%) and clindamycin (75%). Multiplex PCR revealed the SCC*mec* type I as the predominant type in the present study. Our study showed that there was no significant relationship between the presence of different types of *SCCmec* elements and resistance to antibiotics. The present study highlighted a frequent prevalence of MR-CoNS harboring SCC*mec* type genes in Ahvaz, southwest of Iran. Thus, the molecular typing and periodical monitoring of their drug resistance pattern should be considered in national stewardship programs to designing useful antibiotic prescription strategies.

## Introduction

Coagulase-negative staphylococci (CoNS) are gram-positive catalase-positive bacteria, with no ability to clot blood plasma. More than 40 species of CoNS are known as normal flora of the human skin and mucous membranes [1]. In recent years, CoNS have been established as important agents of nosocomial and community-acquired infections especially in patients with medical devices and immunocompromized patients. *Staphylococcus epidermidis* and *Staphylococcus haemolyticus* are identified as the most common species isolated from clinical samples [2].

CoNS are resistant to different antibiotics and can cause several therapeutic problems in public health systems [3]. Various factors, such as biofilm production, the existence of resistant genes, overuse, misuse and inappropriate prescribing of antibiotics, may lead to antibiotic resistance in CoNS [1,4]. In recent years, several studies showed an increase in the incidence of nosocomial infections caused by methicillin resistant CoNS (MR-CoNS) strains [3]. According to the reports, during the 1990s the prevalence of MR-CoNS was higher (75–90%) than methicillin resistant *S. aureus* (MRSA), an observation that continues to be true today [4,5]. Various studies reported a high level of antibiotic resistance against penicillins particularly semi-synthetic penicillins, cephalosporins, macrolides, aminoglycosides and tetracyclines has been observed in CoNS strains [6]. Co-resistance to different classes of antibiotics (especially ciprofloxacin, norfloxacin, gentamicin, nitrofurantoin, erythromycin and amikacin) is higher in MR-CoNS than methicillin sensitive CoNS (MS-CoNS) [7]. Unfortunately, CoNS strains with linezolid resistance and decreased susceptibility to vancomycin have been reported recently [8].

Methicillin-resistance in staphylococci is mediated by the *mecA* gene, which encodes for the penicillin-binding protein 2a (PBP2a) and is harbored in the staphylococcal cassette chromosome *mec* (SCC*mec*), resulting in decreased affinity for the β-lactam antibiotics [7]. Nosocomial infections, due to methicillin resistant staphylococci, are most frequently associated with SCC*mec* types I, II and III that are significantly larger than the other types, while SCC*mec* type IV is mainly related to community-acquired infections [9,10].

Today, CoNS are as important as *S. aureus* in terms of causing significant infections in hospital and community settings. In addition, CoNS have been shown to be a reservoir of SCC*mec* elements. Therefore, phenotypic and genotypic studies (particularly on SCC*mec* properties) are essential for their further characterization and better understanding. The findings can help in infection control, prevention or the development of new antimicrobial agents [1].

As it was mentioned before, prevalence and antimicrobial resistance of CoNS is on the rise [1]. In Iran, the SCC*mec* types have been extensively studied in *S. aureus*, but little is known about their epidemiology in MR-CoNS. Therefore, the present study was designed to evaluate the susceptibility pattern, prevalence and diversity of SCC*mec* types I, II, III and IV in MR-CoNS isolated from patients in southwest of Iran.

## Materials and methods

### Sample collection

In this cross-sectional study from March to August 2018, 90 clinical isolates of CoNS were collected from patients referred to the Golestan teaching hospital (a major referral center placed in a tropical region of southwest Iran). These isolates belonged to different clinical specimens, including urine, wound, blood, catheter tip and sputum as well as different wards, including outpatient department (OPD), intensive care unit (ICU), neonates intensive care unit (NICU), nephrology, surgery, women’s ward and men’s ward.

### Isolation of CoNS

All specimens were cultured on blood agar and MacConkey agar (Merck, Darmstadt, Germany) and incubated aerobically at 37°C for 24 h. Suspected CoNS colonies were identified using standard microbiologic methods including Gram staining, catalase and coagulase tests, and novobiocin susceptibility test [11].

### Phenotypic screening of methicillin resistance

Resistance to methicillin was detected by cefoxitin disc diffusion test using a 30 µg disc (MAST, Berkshire, U.K.). A 0.5 Mc Farland standard suspension of the isolate was made and lawn culture done on Mueller-Hinton agar (MHA) (Merck, Darmstadt, Germany) plates. The plates were incubated at 35°C for 24 h and zone diameters were measured. An inhibition zone diameter of ≤ 24 mm was considered as methicillin resistant and ≥ 25 mm was considered as methicillin sensitive [12]. Methicillin sensitive *S. aureus* (MSSA) ATCC 25923 and methicillin resistant *S. aureus* (MRSA) ATCC 29247 were used as negative and positive controls, respectively. The isolates resistant to Cefoxitin were further tested for the presence of the *mec*A gene.

### Antimicrobial susceptibility testing

Antimicrobial susceptibility testing (AST) was carried out on all MR-CoNS isolates to eight antibiotics by standard disc diffusion method on MHA medium (Merck, Darmstadt, Germany) according to the recommendations of the Clinical & Laboratory Standards Institute (CLSI) [12]. The antimicrobial agents used were clindamycin (2 μg), erythromycin (15 μg), gentamicin (10 μg), trimethoprim-sulfamethoxazole (25 μg), quinupristin-dalfopristin (synercid) (15 μg), rifampin (5 μg), linezolid (30 μg) and ciprofloxacin (5 μg) (MAST, Berkshire, U.K.). Multidrug-resistant (MDR) isolates (resistant to three or more of antimicrobials) were estimated according to previously described definitions [13]. *Staphylococcus aureus* ATCC 29213 and ATCC 33591 were used as control strains.

### Detection of inducible clindamycin resistance

All MR-CoNS isolates that showed resistance to erythromycin and susceptibility to clindamycin in AST were evaluated for inducible clindamycin resistance by D-zone test. The D-zone test was performed by placing a 15 μg erythromycin and 2 μg clindamycin discs at 15–25 mm edge-to-edge distance on Mueller–Hinton agar plates (Merck, Darmstadt, Germany) inoculated by tested isolates. The plates were then incubated at 35 ± 1°C for 18 ± 2 h. The isolates that were showed flattening of the clindamycin inhibition zone adjacent to the erythromycin disc were considered as D-zone test positive (inducible clindamycin resistance), and those with a circular inhibition zone were classified as D-zone test negative [12].

### DNA extraction

DNA extraction was done by the boiling method [14]. The isolates were cultured on nutrient agar (Merck, Darmstadt, Germany) incubated at 37 °C for 24 h. The bacterial suspension was prepared in microtubes containing 500 µl TE (10 mM Tris/HCl, 1 mM EDTA, pH 8.0). The suspension was heated at 95°C for 5 min and then centrifuged at 14,000 rpm for 10 min at 4°C. The supernatant was used as a template for polymerase chain reaction (PCR). Assessment of DNA concentration and quality were performed by measuring the absorbance of A260 and A280 nm with the spectrophotometer and agarose gel electrophoresis, respectively.

### Identification of CoNS species by *tuf* gene

Confirmation of MR-CoNS species was done by amplification and partial sequencing of the *tuf* gene using primers described previously in Table 1 [15]. The *S. epidermidis* ATCC 49134 was used as positive control in the amplification reaction. The obtained sequences of *tuf* gene for each isolate were aligned separately using MEGA 5 (Molecular Evolutionary Genetics Analysis) software and compared with all existing sequences of CoNS annotated in GenBank database (http://www.ncbi.nlm.nih.gov/BLAST). Verified isolates were stored in trypticase soy broth (Merck, Darmstadt, Germany) containing 20% (v/v) glycerol at −70°C for further analysis.

**Table 1:** Primers used in the present study

Primer	Oligonucleotide sequence (5′ to 3′)	Target	Product size (bp)	Reference
tuf-F	GCCAGTTGAGGACGTATTCT	*tuf*	412	[14]
tuf-R	CCATTTCAGTACCTTCTGGTAA			
mecA-F	TCCAGATTACAACTTCACCAGG	*mecA*	162	[15]
mecA-R	CCACTTCATATCTTGTAACG			
1272F1	GCCACTCATAACATATGGAA	*IS1272*	415	[16]
1272R1	CATCCGAGTGAAACCCAAA			
β	ATTGCCTTGATAATAGCCYTCT	*ccrA2-B*	937	[16]
α3	TAAAGGCATCAATGCACAAACACT			
IS1272	CGTCTATTACAAGATGTTAAGGATAAT	*ccrC*	518	[16]
ccrCR	CCTTTATAGACTGGATTATTCAAAATAT			

### Detection of the *mecA* gene and SCC*mec* typing

Detection of the *mecA* gene was performed by polymerase chain reaction (PCR), using primers described previously (Table 1) [16]. SCC*mec* typing of isolates was determined by multiplex PCR described by Boye et al [17]. The 50-µl PCR mixtures consisted of 1× AmpliTaq PCR buffer (SinaClon, Tehran, Iran), 1.5 mM MgCl_2_, 200 µM of each dNTP and 1 U of AmpliTaq DNA polymerase (SinaClon, Tehran, Iran). Primer concentrations were as follows: primers β and α3, 0.2 µM each; ccrCF and ccrCR, 0.25 µM each; 1272F1 and 1272R1, 0.08 µM each. The primers sequences used for the SCC*mec* typing are shown in Table 1. The PCR assay was carried out in an Eppendorf thermocycler (Roche Co., Mannheim, Germany), with following Protocol: initial denaturation at 94°C for 4 min, followed by 30 cycles of denaturation at 94°C for 30 s, annealing at 55°C for 30 s, extension at 72°C for 60 s and a final extension at 72°C for 4 min. The PCR products (5 µl) were separated by electrophoresis (80 V, 40 min) using a 1% agarose gel (Sinaclon, Tehran, Iran) in 1× TBE buffer containing DNA safe stain (1:10,000 dilution in TBE) (Sinaclon, Tehran, Iran) and then visualized using a Gel doc UV illuminator system (Proteinsimple, San Jose, CA, U.S.A.). *S. aureus* ATCC 29247 strains (for *mecA* gene), *S. aureus* NCTC 10442 (for Type I), *S. aureus* N315 (for Type II), *S. aureus* 85/2082 (for Type III), *S. aureus* JCSC 4744 (for Type IV) and the sterile deionized water were included with each PCR run as positive and negative controls, respectively.

### Statistical analysis

The analysis was performed by using SPSS™ software, version 22.0 (IBM Corporation, Armonk, NY, U.S.A.). The results are presented as descriptive statistics in terms of relative frequency. Values are expressed as the percentages of the group (categorical variables). Chi-square or Fisher’s exact tests were used to determine the significance of differences. A difference was considered statistically significant if the *P*-value was < 0.05.

## Results

### Frequency of MR-CoNS

In the present study based on standard biochemical and microbiological tests, 90 CoNS were isolated, of which 44 isolates were confirmed as MR-CoNS by the disc diffusion and PCR of *mecA* gene methods. Phenotypic (cefoxitin disc) and molecular (*mecA* detection) based MR-CoNS screening methods showed similar results. Twenty-three (52.3%) MR-CoNS belonged to females and twenty-one (47.7%) belonged to males. All collected CoNS were as single isolate of clinical samples. The most common source of species was urine (*n* = 30, 68.2%) followed by wound (*n* = 6, 13.6%), blood (*n* = 3, 6.8%), catheter tip (*n* = 3, 6.8%) and sputum (*n* = 2, 4.5%). Isolates were obtained from different wards, including OPD clinics (*n* = 25, 56.8%), ICU (*n* = 5, 11.4%), Women’s ward (*n* = 5, 11.4%), NICU (*n* = 4, 9.1%), surgery (*n* = 2, 4.5%), nephrology (*n* = 2, 4.5%) and Men’s ward (*n* = 1, 2.3%) (Table 2). The frequency of various MR-CoNS species demonstrated by PCR amplification of *tuf* gene followed by DNA sequencing revealed that the predominant species of MR-CoNS were *S. epidermidis* and *S. haemolyticus* each (*n* = 20, 45.4%) followed by *S. hominis* (*n* = 2, 4.5%), *S. saprophyticus* and *S. petrasii* each (*n* = 1, 2.3%), respectively.

**Table 2:** Distribution of 44 MR-CoNS isolates in clinical specimens and hospital wards

Bacterial species	MR-CoNS	*S. haemolyticus*	*S. epidermidis*	*S. hominis*	*S. saprophyticus*	*S. petrasii*
	No. (%)	20 (45.5)	20 (45.5)	2 (4.5)	1 (2.3)	1 (2.3)
**2a. Distributed of MR- CoNS isolates from clinical specimens**						
Clinical specimens	Wound	2 (4.5)	3(6.8)	0 (0)	0 (0)	1 (2.3)
	Urine	14 (31.8)	13 (29.5)	2 (4.5)	1 (2.3)	0 (0)
	Blood	2 (4.5)	1 (2.3)	0 (0)	0 (0)	0 (0)
	Catheter tip	2 (4.5)	1 (2.3)	0 (0)	0 (0)	0 (0)
	Sputum	0 (0)	2 (4.5)	0 (0)	0 (0)	0 (0)
**2b. Distributed of MR-CoNS isolates from hospital wards**						
Hospital wards	Women’s ward	1 (2.3)	2 (4.5)	1 (2.3)	0 (0)	1 (2.3)
	Men’s ward	1 (2.3)	0 (0)	0 (0)	0 (0)	0 (0)
	ICU	2 (4.5)	3 (6.8)	0 (0)	0 (0)	0 (0)
	Surgery	3 (6.8)	1 (2.3)	0 (0)	0 (0)	0 (0)
	OPD	11 (25)	12 (27.2)	1 (2.3)	1 (2.3)	0 (0)
	NICU	1 (2.3)	1 (2.3)	0 (0)	0 (0)	0 (0)
	Nephrology	1 (2.3)	1 (2.3)	0 (0)	0 (0)	0 (0)

### Antibiotic resistance patterns

According to the results of disc diffusion susceptibility testing, the highest resistances were observed to erythromycin (84.1%), followed by clindamycin (75%), gentamicin (65.9%), rifampin and ciprofloxacin (59.1%), trimethoprim-sulfamethoxazole (52.3%), synercid (34.1%) and linezolid (22.7%). On the other hand, the most effective antibiotics were linezolid (77.3%) and quinupristin–dalfopristin (63.2%), respectively (Table 3). Among 44 MR-CoNS strains, only 2 (4.5%) isolates including *S. haemolyticus* and *S. hominis* showed the inducible clindamycin resistant phenotype by D-zone test.

**Table 3:** Antibiotic susceptibility profile of 44 MR-CoNS isolates

Antibiotic	Number (%)		
	Susceptible	Intermediate	Resistant
Rifampin	11 (25)	7 (15.9)	26 (59.1)
Clindamycin	9 (20.5)	2 (4.5)	33 (75)
Erythromycin	4 (9.1)	3 (6.8)	37 (84.1)
Gentamycin	15 (34.1)	0 (0.0)	29 (65.9)
Trimethoprim–sulfamethoxazole	21 (47.7)	0 (0.0)	23 (52.3)
Ciprofloxacin	13 (29.5)	5 (11.4)	26 (59.1)
Linezolid	34 (77.3)	0 (0.0)	10 (22.7)
Synercid	28 (63.6)	1 (2.3)	15 (34.1)

### Multi-drug resistance profiles

According to the AST, 34 (77%) of 44 MR-CoNS isolates that were resistant to at least three different classes of antimicrobial agents considered as MDR, with 20 different patterns (Table 4). The pattern 1 (rifampin–gentamycin–erythromycin–clindamycin–ciprofloxacin) with a frequency rate of 18.8% was the most prevalent resistance profile (Table 4).

**Table 4:** Multidrug-resistance patterns of 44 MR-CoNS isolates

Resistance pattern	Phenotypic resistance	Number of resistant MR-CoNS isolates (%)
1	Rif- Gm- ERY- CM- CP	8 (18.8)
2	Rif- Gm- ERY- CM- CP- TS	4 (9.1)
3	Gm- ERY- CM- CP- TS	3 (6.8)
4	Gm- ERY- CM- CP- SYN	2(4.5)
5	Rif- Gm- ERY- CM- CP- SYN	2(4.5)
6	Rif- ERY- CM- LZD- SYN- TS	2(4.5)
7	Rif- ERY- CM- LZD	1(2.3)
8	Gm- ERY- CP- LZD	1(2.3)
9	Rif- Gm- ERY- CM- SYN	1(2.3)
10	Rif- ERY- CM- TS	1(2.3)
11	Rif- Gm- ERY- CM- TS	1(2.3)
12	Rif- Gm- CM- CP- TS	1(2.3)
13	Gm- ERY- CM- CP- LZD- TS	1(2.3)
14	ERY - SYN- TS	1(2.3)
15	Gm- ERY- CM- SYN- TS	1(2.3)
16	Rif- Gm- ERY- CM- Cp- TS- SYN	1(2.3)
17	Rif- Gm- ERY- CM- LZD- TS- SYN	1(2.3)
18	ERY- CM- CP- LZD- TS- SYN	1(2.3)
19	Gm- ERY- CM- Cp- LZD- TS- SYN	1(2.3)
20	Rif- Gm- ERY- CM- Cp- LZD- TS- SYN	1(2.3)

Abbreviations: CM, clindamycin; CP, ciprofloxacin; ERY, erytomycin; Gm, gentamycin; LZD, linezolid; Rif, rifampin; SYN, synercid; TS, trimethoprimsulfamethoxazole.

### PCR amplification of the *mecA* gene and SCC*mec* typing

The presence of *mecA* was confirmed in 44 MR-CoNS isolates by PCR. According to our results 27 isolates showed single type, including type I (*n* = 20, 45.5%), type IV (*n* = 4, 9.1%), type III (*n* = 2, 4.5%) and type II (*n* = 1, 2.3%). Six isolates had two types, III+ I (*n* = 5, 11.4%) and IV+ III (*n* = 1, 2.3%). Eleven (25%) isolates showed no band for SCC*mec* I-IV (Table 5). The 11 MR-CoNS isolates that lacking SCC*mec* I-IV were tested by single-plex PCR for the four loci, but no SCC*mec* types were detected in these isolates. Fifty-five percent of SCC*mec* type I positive MR-CoNS isolates (11/20) belonged to *S. haemolyticus* species. The assessment of association between presence of different SCC*mec* types and antibiotic resistance in 44 MR-CoNS isolates showed that there was no significant relationship (*P* > 0.05) (Table 6).

**Table 5:** Distribution of SCC*mec* elements among 44 MR-CoNS isolates

SCCmec type	Total No. (%)	*S. epidermidis*	*S. haemolyticus*	*S. hominis*	*S. saprophyticus*	*S. petrasii*
I	20 (45.5)	7 (15.9)	11 (25)	1 (2.3)	0 (0)	1 (2.3)
II	1 (2.3)	0 (0)	1 (2.3)	0 (0)	0 (0)	0 (0)
III	2 (4.5)	1 (2.3)	1 (2.3)	0 (0)	0 (0)	0 (0)
IV	4 (9.1)	4 (9.1)	0 (0)	0 (0)	0 (0)	0 (0)
I and III	5 (11.4)	2 (4.5)	3 (6.8)	0 (0)	0 (0)	0 (0)
III and IV	1 (2.3)	0 (0)	1 (2.3)	0 (0)	0 (0)	0 (0)
Lacking SCCmec I-IV	11 (25)	6 (13.6)	3 (6.8)	1 (2.3)	1 (2.3)	0 (0)

**Table 6:** Association between SCC*mec* types and antimicrobial resistance patterns of 44 MR-CoNS isolates

Type of SCC*mec*	Rif No. (%)	Gm No. (%)	ERY No. (%)	CM No. (%)	CP No. (%)	LZD No. (%)	SYN No. (%)	TS No. (%)
I	12 (27.2)	14 (31.8)	17(38.6)	15 (34)	11 (25)	2 (4.5)	6 (13.6)	9 (20.4)
II	0 (0)	0 (0)	1 (2.3)	0 (0)	0 (0)	0 (0)	1 (2.3)	1 (2.3)
III	2 (4.5)	2 (4.5)	2 (4.5)	2 (4.5)	1(2.3)	0 (0)	1 (2.3)	1 (2.3)
IV	2 (4.5)	2 (4.5)	3 (6.8)	2 (4.5)	2 (4.5)	1 (2.3)	2 (4.5)	1 (2.3)
*P* value	0.385	0.651	0.919	0.459	0.675	0.330	0.261	0.453

Abbreviations: CM, clindamycin; CP, ciprofloxacin; ERY, erytomycin; Gm, gentamycin; LZD, linezolid; Rif, rifampin; SYN, synercid; TS, trimethoprim–sulfamethoxazole.

## Discussion

MR-CoNS are one of the most important causative agents of human infections in which the MDR property is more visible than the MS-CoNS [18]. Owing to the paucity of information about the frequency of SCC*mec* types among various MR-CoNS strains in the southwest of Iran, the present study aimed to investigate the rate of MR-CoNS in this region. The prevalence of MR-CoNS in clinical samples has been reported between 47% and 91% in different cities of Iran [19,20,21,22]. The rate of methicillin resistance in our study was 48.8% that was close to Namvar et al. [19] and Hajiahmadi et al. [23] results with 47.2% and 50%, respectively.

In our study, the most common MR-CoNS isolates belonged to both of *S. epidermidis and S. haemolyticus* (45.45%) which was consistent with previous reports from Iran and other countries [3,23,24,25]. One of the species that so far has not been reported from other regions of Iran, but was isolated in Ahvaz city, was *S. petrasii*. *Staphylococcus haemolyticus* was the predominant isolate from urine specimens in our study (31.8%). Our results confirmed the results of previous studies in which the most common species isolated from urine was *S. haemolyticus* [3,26]. In the present study resistance to eight antibiotics was considered. The highest resistance was observed to erythromycin (84.1%). Nahaei et al. closest to our findings showed the high rate of resistance to erythromycin among MR-CoNS (84.8%) [21].

In the present investigation, the susceptibility patterns of MR-CoNS were at variance with some other studies from Iran. For example in Hajiahmadi et al. [23] and Havaei et al. [27] studies the highest resistance was observed to trimethoprim–sulfamethoxazole (56%) and ciprofloxacin (65%) respectively. This could be because of different protocols and panels of antibiotics used in various hospitals. Although in our study the lowest resistance was observed to linezolid (22.7%), this rate of resistance was high in comparison with many studies in Iran and other countries [23,28,29,30]. According to multinational and multicenter surveillance studies, more than 99% of coagulase-negative staphylococci and *S. aureus* clinical strains are sensitive to linezolid (31) [31]. The common mechanisms for linezolid resistance are mutations in the linezolid *23S rRNA* binding site and acquisition of a plasmid-borne ribosomal methyltransferase gene called the *cfr* (chloramphenicol/florfenicol resistance) gene. Later is more worrisome because of its rapid spread and possibility of its transfer to more pathogenic organisms, such as *S. aureus* [8]. The cfr enzyme causes resistance to several other antibiotic classes besides oxazolidinones, such as phenicols, lincosamides, pleuromutilins and streptogramin compounds [32]. In different countries such as Italy, Spain, USA, Mexico, and China, the emergence of *cfr* in nosocomial staphylococci has been reported [32,33]. Additionally, the possibility of *cfr* gene transmission from veterinary isolates, such as *S. sciuri, S. warneri, S. aureus, S. hyicus* and *Enterococci* has been reported [34]. Because the linezolid isn’t used routinely as a common antibiotic in our region, it is difficult to accept this percentage of resistance. Therefore, for a better understanding of high resistance reason to linezolid and some other antibiotics, more studies about the existence of *cfr* gene among CoNS can be helpful. Moreover, the result of inducible resistance to clindamycin assessed by D-test was 4.5%. There is little information about the rate of inducible resistance to clindamycin among MR-CoNS in Iran. Aghazadeh et al. [29] and Abdollahi et al. [35] closest to our findings showed the rate of inducible clindamycin resistance among MR-CoNS 6% and 5% respectively. According to Emaneini et al. [36] results, 14.1% of MR-CoNS had inducible resistance to clindamycin.

In the present study, SCC*mec* typing permitted the differentiation of types I–IV among the MR-CoNS strains. The SCC*mec* is a mobile genetic element widely distributed among MR-CoNS species that varies depending on the host species, various environments and geographical locations [16]. In the present study, the most abundant SCC*mec* type among MR-CoNS was type I (45.5%), whereas in other studies in Iran SCC*mec* type IV, III and V were more frequent [20,23,28]. It isn’t easy to compare our results with other results in Iran because in many studies the diversity of SCC*mec* type has been evaluated only among *S. epidermidis* [19,23,28]. Additionally, the high percentage of SCC*mec* type V among *S. epidermidis* in some studies can be associated with the presence of a large number of commensal samples, among which a higher prevalence rate of SCC*mec* type V was proven [37]. Different countries have reported varied SCC*mec* types in MR-CoNS. For example, SCC*mec* type I has been reported to be the most predominant in India, whereas SCC*mec* types II and III have been found to be the most common in Nigeria and China respectively [25,38]. In our study, six isolates had two types: III+ I (*n* = 5, 11.4%) and IV+ III (*n* = 1, 2.3%). This was not surprising as the co-existence of two SCC*mec* elements appears to be common in MR-CoNS [25,39,40]. In our study, carriage of SCC*mec* elements was not associated with resistance to tested antibiotics. In a study by Garza-Gonzalez et al. [24], a significant correlation between the presence of the SCC*mec* type III and resistance to meropenem (*P*<0.05) was reported. Machado et al. [40] showed that isolates with SCC*mec* type III were more resistant to non-β-lactam antimicrobials than isolates with other SCC*mec* types, although the increase in resistance was statistically significant only for clindamycin (*P* = 0.021), rifampicin (*P* = 0.010) and levofloxacin (*P* = 0.005).

To date, more than ten SCC*mec* types have been reported in CoNS [25]. A limitation of our study was that we only tried to search for the most common SCC*mec* types in Iran. Our PCR targeted only SCC*mec* types I–IV, but 11 isolates in this study were negative for these four types indicating that the MR-CONS isolates in our region have SCC*mec* types belonging to the other established types or have novel types. Generally, performing diverse typing methods can afford better and more valuable epidemiological data about the clonal diversity of the isolates that can be advantageous for outbreak researches [41,42]. Another limitation of the present study was the lack of determination of the minimum inhibitory concentrations of tested antibiotics.

## Conclusion

Our study was the first study for the determination of frequency and diversity of SCC*mec* types in MR-CoNS isolates in Ahvaz, Iran. The present study highlighted a frequent prevalence of multidrug-resistant CoNS harboring SCC*mec* type genes. The SCC*mec* type I was the most common in Ahvaz city, southwest of Iran. Since CoNS are important causes of nosocomial and community-acquired infections especially in patients with medical devices, molecular typing and periodical monitoring of their drug resistance pattern should be considered in national stewardship programs to designing useful antibiotic prescription policies and hospital infection control strategies.

## Abbreviations

AST: antimicrobial susceptibility testing
CoNS: coagulase-negative staphylococci
MDR: multidrug-resistant
MR-CoNS: methicillin resistant coagulase-negative staphylococci
